# An Optimization Tool to Formulate Diets within a Supplementary Nutrition Program for Children^☆^

**DOI:** 10.1016/j.cdnut.2024.104409

**Published:** 2024-06-29

**Authors:** Fathima Ayoob, Jawahar R Manivannan, Ashikh Ahamed, Afsal K Murikkanchery, Ankita Mondal, Gowri Bhatnagar, Melari S Nongrum, Sandra Albert, Pulkit Mathur, Lalita Verma, Radhika Madhari, Srirangam A Brinda, Suparna Ghosh-Jerath, Vanisha Nambiar, Hemangini Gandhi, Syed Z Quazi, Rachita Gupta, Harshpal S Sachdev, Anura V Kurpad, Tinku Thomas

**Affiliations:** 1 Division of Epidemiology & Biostatistics, St John’s Research Institute, Bengaluru, Karnataka, India; 2 Indian Institute of Public Health Shillong, Shillong, Meghalaya, India; 3 Department of Food and Nutrition and Food Technology, Lady Irwin College, University of Delhi, New Delhi, Delhi, India; 4 Department of Dietetics Division, National Institute of Nutrition, Osmania University, Hyderabad, Telangana, India; 5 Department of Nutrition, The George Institute for Global Health India, New Delhi, Delhi, India; 6 Department of Foods and Nutrition, Maharaja Sayajirao University of Baroda, Vadodara, Gujarat, India; 7 Datta Meghe Institute of Higher Education and Research, Wardha, Maharashtra, India; 8 World Health Organization Country Office for India, New Delhi, Delhi, India; 9 Pediatrics and Clinical Epidemiology, Sitaram Bhartia Institute of Science and Research, New Delhi, Delhi, India; 10 Department of Physiology, St. John’s Medical College, Bengaluru, Karnataka, India; 11 Department of Biostatistics, St John’s Medical College, Bengaluru, Karnataka, India

**Keywords:** supplementary nutrition program, optimization tool, linear programming, ICDS, Schildren less than 6 years

## Abstract

**Background:**

In large supplementary feeding programs for children, it is challenging to create and sustain contextual, acceptable, nutritionally complete, and diverse supplemental foods. For example, the Indian Supplementary Nutrition Program (SNP) supplements the dietary intake of children, pregnant and lactating women, and severely acutely malnourished (SAM) children by offering dry take home rations (THRs) or hot cooked meals (HCMs) across India, but an optimization tool is necessary to create local contextual recipes for acceptable and nutritionally adequate products.

**Objectives:**

This study aimed to create a linear programming (LP) model to optimize diverse food provisions for a SNP to meet its program guidelines, using locally available foods, within budgetary allocations.

**Methods:**

A LP algorithm with appropriate constraints was used to generate an optimal THR based on raw foods, or an optimal weekly HCM menu comprised of a lunch meal with mid-morning snacks, based on user choices of foods and recipes. The database of foods used was created by a prospective survey conducted across all states of India for this purpose, such that the recipe and food optimization was diverse and specific to the guidelines for each beneficiary group.

**Results:**

An interactive web-based app, which can optimize feeding programs at any population level, was developed for use by program implementers and is hosted at https://www.datatools.sjri.res.in/SNP/. In the Indian example analyzed here, the recommended optimized diets met the guidelines for diversified and nutritionally complete SNP provision but at a cost that was almost 25% higher than the present Indian budget allocation.

**Conclusions:**

The optimization model developed demonstrates that contextual SNP diets can be created to meet macronutrient and most essential micronutrient needs of large-scale feeding programs, but appropriate diversification entails additional costs.

## Introduction

Diets of children and their mothers are often inadequate across most low- and middle-income countries. A key characteristic of these diets is that they are cereal dominated, with a lack of diet diversity, which is a key indicator of the daily nutritional sufficiency that is essential for promoting optimal health and function [1]. For example, in India, the proportion of children aged 6 to 23 mo having minimum diet diversity, or a minimum acceptable diet, remains very low at 25% and 11%, respectively [1]. Some form of whole food supplementation is often required as public health nutrition policy for these children, either as take home food baskets or dry rations, or as cooked meals provided in facilities. Implementing these policies that supply diverse foods beyond cereals, in different forms, to poor populations can have a significant role in tackling malnutrition and promoting dietary diversity [2] for greater health and function. However, different countries (and even subregions within countries) have different supplementary feeding policies depending on their needs, funds, and local foods available. Pulling these together in a coherent, sustainable, region-specific, and cost-sensitive manner requires some form of diet modeling with respect to all the contextual variables.

The Integrated Child Development Services (ICDS) scheme of India is one such example of a multipronged program with supplementary nutrition as an integral activity. The core objective of the program is to decrease the prevalence of undernutrition and associated issues through a unified and outcome-focused strategy [2]. The Supplementary Nutrition Program (SNP), carried out through ICDS centers called *Anganwadis*, provides food for normal and undernourished children, as well as pregnant and lactating mothers, as take home rations (THRs) or hot cooked meals (HCMs). These provisions differ in composition across different Indian states but typically consist of cereals like rice and wheat, pulses like lentils or soybean, along with oil and sugar. In some instances, milk and egg are included. For children, the SNP supplies 500 kcal and 12 to 15 g protein/d through each THR (younger children) or HCM (older children). This has recently been revised to 400 kcal, 15 to 20 g fat/d, 15 to 20 g total protein/d along with some micronutrients [3]. Fulfilling these revised SNP guidelines using natural foods is challenging and needs computing assistance. It is also important to identify the nutrients that cannot be met even through these optimized diets that may require chemical supplementation.

Linear programming (LP) is an effective tool for formulating nutritionally sound food-based recommendations within a limited budget [4]. With support from WHO-India, we aimed to develop a LP-based algorithm with an interactive tool to optimize the raw food provisions for THRs or HCMs, utilizing locally available foods that aligned with regional preferences while adhering to the nutritional rules and budget set by the Indian Government. The process of development of the optimization tool for the ICDS-SNP program in this instance (ICDS-SNP tool), and the demonstration of its ability to meet nutrient recommendations within budgetary constraints, are explored in this article.

## Methods

The ICDS-SNP tool was developed to optimize raw local food provisions to meet the nutritional rules for THRs or HCMs for all categories of beneficiaries, such as children, pregnant and lactating women, as well as children with severe acute malnutrition (SAM). As a working example in this report, the optimization was conducted for feeding children from 6 mo to 6 y, but the tool can be used for any beneficiary. The tool was also developed for use by state and district level officials responsible for selecting and procuring the foods that make up the THRs and for preparing weekly HCM menus. Beyond meeting nutrient recommendations, the tool also facilitated the development of contextually diverse diets.

### Data for optimization

Local raw food data were collected from all 28 states of India and 2 union territories. In each state, 2 districts each were purposively selected, and from them, 2 Anganwadis each were chosen. The Anganwadi staff and district officials responsible for the menu and diet plans of the SNP were interviewed using structured questionnaires. The sampling followed is provided in Supplemental Figure 1. Data on current practices, such as the structure of the weekly menu for HCMs, their raw ingredient quantities, serving size of prepared foods, THR ingredients, fortified foods used, the use of other nutritionally dense foods such as eggs and milk, market prices, the availability of alternate and seasonal foods for THRs and HCMs, and budget allocation for different age groups were collected from the Anganwadi staff. Additional information, such as other foods given to beneficiaries, and foods provided through other schemes (other than the SNP), were also collected. The mothers of 10 children attending each sampled Anganwadi were interviewed with a structured questionnaire to obtain an additional list of local foods that were considered nutritious and fed to children, through a 3-d diet recall. This data was to expand the list of foods to be considered for optimization to more nutritious and locally acceptable foods. The cost of food is critical for optimization. Therefore, a market within the vicinity of the Anganwadi was also chosen, and shopkeepers were interviewed on local and seasonal prices of fruits and vegetables listed either at the Anganwadi or by the mothers of beneficiaries. A database of foods/recipes and the cost of their ingredients was thus developed.

All data were collected by 6 regional centers led by a senior nutritionist (from August 2022 to February 2023) to cover the states in the South, North, West, East, Northeast, and Central regions of India. The cooked food recipes collected for each state from the Anganwadis and their beneficiaries were standardized for their ingredients and quantities by the regional centers. The data collected may have several methods of preparation of one particular food, for example: a lentil curry. The regional centers reviewed all the recipes collected in their center and identified a standard set of ingredients and preparation of each recipe, which was then considered a standard recipe. This also involved improving the nutritive value of some of these recipes by changing some ingredients (e.g., marginally increasing the oil used in preparation to improve the fat content of the recipe). This exercise also ensured that implausible quantities and ingredients were removed. In addition, new recipes that were nutritious and suitable for complementary feeding, as suggested by the regional centers, were considered. Each regional center tested and verified different types of recipes (breads, rice, gravies, vegetable stir fries, snacks, etc.) for ingredient quantity and final product quantity by weighing and cooking these recipes using standard utensils and measuring spoons as they were most familiar and knowledgeable about the regional recipes and their variations. The data on serving sizes of the different foods collected from the Anganwadi centers were also verified as part of the standardization. Multiple HCM recipe combinations such as “steamed rice and green gram lentil gravy” or “steamed rice and ladies finger curry” were then made by the regional centers based on local dietary practices. The combinations of these recipes served as the database from which the optimization was performed, restricted to the combinations of recipes with raw foods chosen by the user. All centers were trained on electronic data collection and recipe standardization using manuals and standard operating procedures prepared for the project by a project coordinating team. Periodic monitoring visits and calls were performed by the project coordinating team to ensure quality of data collection. All data were collected into a database management program developed specifically for the project by the coordinating team, and the data were monitored in real time.

The nutritive value for each raw food ingredient in the database was obtained from Indian Food Composition Tables, IFCT 2017 [5]. The nutritive values of raw foods not listed in the IFCT were obtained from USDA tables [6]. The nutrients considered were energy (kilocalories), protein (grams), fat (grams), dietary fiber (grams), calcium (milligrams), zinc (milligrams), iron (milligrams), magnesium (milligrams), vitamin A (micrograms), folate (micrograms), vitamin B_12_ (micrograms), vitamin B_1_ (milligrams), vitamin B_2_ (milligrams), vitamin B_3_ (milligrams), vitamin B_6_ (milligrams), and vitamin C (milligrams).

A state-specific database of recipes with their ingredients and nutritive values (for the nutrients listed above), as well as cost per serving, was prepared for the HCMs. The food groups considered were cereals and millets, pulses and legumes, green leafy vegetables, other vegetables, roots and tubers, fruits, egg, milk and milk-based products, nuts and dry fruits, and sugars. This allows users to choose from recipes tailored to their region, based on produce available for their Anganwadis. Similarly, for the THR, a database of raw foods, with price per kilogram of food, was prepared for the tool.

### LP model

LP was performed for nutrient allocation toward optimizing a linear objective function, conditional on a set of linear inequalities or equations, referred to as constraints. These constraints defined the boundaries and limitations of the problem being solved. The objective function, which represented the quantity to be maximized or minimized, aligned with the optimization goal. The main objective of the LP model was to identify optimal food quantities that minimize the cost of the diet, conditional on meeting the nutrient recommendations and maintaining diet diversity. Thus,

$$Minimize\;Total\;Cost=\min\;\{\;\sum_{i=1}^{I}Q_{i}\times C_{i}\;\}$$

Where, for HCM, $Q_{i}$ is the serving size of recipe *i*, and $C_{i}$ is the cost per serving size of recipe *i*. For THR, $Q_{i}$ is the quantity of raw food item i (in grams) and $C_{i}$ is the cost per gram of raw food item i. The minimized cost is the cost of provision of an HCM or THR per beneficiary per day.

The primary constraints for the model were the recommendations for energy, protein, and fat specified by the guidelines of the program (Table 1)—in this case, the ICDS rules for the specific beneficiary groups [3]. An additional constraint was for the maximum safe daily nutrient intake, called the tolerable upper limit [7] for all micronutrients listed in the guidelines. For children with SAM, for whom the recommendations were higher, the model provided an additional 1 or 2 snacks, slightly increased the quantity of the optimized HCM, or both to meet the recommendation.

**TABLE 1:** The Government of India guidelines for macronutrient content in the ICDS-SNP

Nutrients	Child			SAM child			Pregnant women	Lactating women
	6–12 mo	1–3 y	3–6 y	6–12 mo	1–3 y	6–12 mo		
Energy, kcal	200	400	400	400	700	800	600	600
Protein, g	8	15	15	15	25	25	22	22
Fat, g	10	15	15	15	25	25	22	22

Abbreviation: ICDS, Integrated Child Development Services; SAM, severe acute malnutrition; SNP, Supplementary Nutrition Program.

For the HCM weekly menu optimization, the recipes were classified into 3 categories: *1*) main dish, *2*) curry to accompany the main dish, and *3*) side dish. The lower limit for serving size for each recipe category was predetermined, based on the quantity a child could reasonably consume at one sitting. The optimized quantity of food could be a proportion of this serving size and ≤1.5 times this quantity, which was set as the maximum allowable quantity that could be recommended for a given recipe. In addition to the recipe categories mentioned above, some foods were marked as “compulsory” or “additional” foods. The “additional” foods were those that complemented various dishes and could be paired with any food, like chutneys and salads. The “compulsory” foods are chosen before the optimization, and the foods that can be considered as compulsory are egg, milk, and milk powder, which if selected will be mandatorily included in the optimized solution with the optimized quantity along with other foods. The selected “additional” food will be part of the optimization model, however it may or may not be present in the final solution. The “additional” food could also be opted by the user after the optimization is performed. This will provide additional nutritive value for the weekly menu if it can be accommodated within the budget.

For the THR, a constraint on the inclusion of ≥1 food item from each food group (cereals, pulses and legumes, sugars, and edible oils and fats) was specified. Furthermore, constraints on the minimum and maximum quantities within each food group were set for whole foods and blended premixes separately. Similarly, certain ingredients (oils, eggs, ground nuts, sugar, jaggery, whole milk powder, black and white sesame seeds) were subjected to quantity restrictions at the food item level. For example, a 3- to 6-y-old child could receive a blended premix containing oil ranging from 5 to 10 g. Additionally, a consistent cereal to pulse ratio of 2:1 was to be maintained for the THR as this is part of the ICDS-SNP guideline. Finally, an optional constraint was added to the optimization model, enabling the user to compulsorily include a cereal or millet in their THR product if desired.

All the optimization constraints considered in the model are provided in the Supplementary Material.

### Optimization tool

The LP algorithm described above for the THR and HCM of each state of India was converted into a user-friendly, interactive web-based tool to suggest multiple context-specific food combinations with optimal nutrient provision at minimal cost. If the optimal solution with a set of foods chosen by the user was not achievable within the given budget available, in this instance, the ICDS budget of ₹8 per child per day, the model would provide the next least expensive solutions based on chosen foods that fulfilled all the nutrient guidelines (Table 1). This step allowed the assessment of the affordability of the options to find the most suitable solution that aligned with budgetary limitations. Thus, users could create a weekly menu by combining or repeating several of the suggested meals. All these features are available for any user of the tool.

Additionally, a user could register (a free option) and have login credentials to make alterations to the database of the tool for his/her use. The features available for a registered user are options to add new recipes and ingredients to the database, thereby customizing the database for the user, or to alter specified nutrient recommendations against which the optimization was constrained. For example, a registered user could add a vegetable and recipes with that vegetable if it is not already part of the database. The user could then opt for this new vegetable when he/she runs the optimization. If the ICDS guideline changes or a particular state decides to have some difference in the guidelines, a registered user can make changes to the guidelines within the tool such that the linear program considers new constraints for his/her use. This feature makes the tool relevant for both HCMs and THRs even when government recommendations can change. The changes or additions made by registered users will be available for their future optimization but will not be available for others.

The market prices specific to each Indian state were gathered from local retail markets, and the subsidized cost of ingredients were obtained from ICDS officials. State governments have the freedom to subsidize the price of some food items for use in ICDS such that the food is procured for ICDS at a rate lower than that in the retail market. The user can enter the subsidized cost in the tool and consequently, the subsidized prices of these foods will be considered for the optimization within those states.

Users can modify the cost of each raw food ingredient, based on the actual price of procurement of foods, using the tool. Registered users can also save the revised rates in the database for future reference. Because certain ingredients are universally and mandatorily fortified by government programs, these foods have been considered as fortified [8] in the tool, but the user has the option to change this if required (Supplemental Table 1). Finally, a feature to compulsorily include a certain food, like millets or cereals for a THR product, was also included.

The model and the ICDS-SNP tool were applied to optimize the HCMs and THRs for children in the Indian state of Karnataka as an example, choosing foods listed in Supplemental Tables 2 and 4 and the recipe database for this state. Detailed documentation on the optimization and construction of the tool (https://www.datatools.sjri.res.in/SNP/img/Documents/WHO_Documentation.pdf),as well as a detailed audio (https://youtu.be/ryUACZ4eydM, https://youtu.be/jrEkoQr186M) and text manual (https://www.datatools.sjri.res.in/SNP/img/Documents/User_Manual_WHO.pdf) for the use of the tool for THRs and HCMs are also available. In this instance, the tool has options to optimize for all the SNP beneficiaries in the ICDS, including children with SAM, for whom the recommendations for nutrients are different, but with appropriate databases, it can be used anywhere.

## Results

When the ICDS-SNP tool was used to optimize a weekly HCM diverse diet plan for 3- to 6-y-old children in the state of Karnataka, the mean (SD) cost per day for a child, satisfying the ICDS recommendations, was ₹9.6 (SD 0.145) based on the data provided in Supplemental Table 2, which was higher than the budgeted funds available of ₹8 per day per child ($0.09). The excess cost of the optimized solution was because of the compulsory inclusion of eggs, necessary for meeting some of the nutrient recommendations, among other foods. However, there were ways to reduce this cost if food subsidies were available; for example, if the cost of egg could be subsidized from ₹5 to 2.5; this could reduce the HCM price to ₹7.2 per child per day, which was lower than the budgeted ₹8. Supplemental Table 2 shows the optimized menu for HCM in Karnataka with a certain choice of foods, along with the retail market price and subsidized prices. The optimized solution met the recommendations for all macronutrients as well as for micronutrients such as iron, zinc, folate, magnesium, and vitamin A for children aged 3 to 6 y (Table 2). However, the optimized diet could not meet calcium (74%) and vitamin B (53%–87%) recommendations. Thus, although the tool is mandated to meet close to 100% of the macronutrient guidelines without optimizing the micronutrients, during the process of fulfilling macronutrients, most of the micronutrient guidelines are also met, and the tool calculates and displays the micronutrient provision from the optimized meal for further reference and improvement.

**TABLE 2:** Nutrient composition in an example of a cost-optimized HCM for children aged 3 to 6 y in the Indian state of Karnataka

Nutrient	Guidelines	Nutrient content	Percentage of guidelines	Percentage of guidelines^1^
Energy, kcal	400.0	400.1	100.0	102.0
Protein, g	15.0	17.0	114.0	95.0
Fat, g	15.0	14.0	94.0	94.0
Calcium, mg	150.0	110.8	74.0	143.0
Dietary fiber^2^, g	6.8	5.1	76.0	74.0
Folate, μg	40.0	75.2	189.0	148.0
Iron, mg	3.0	3.7	125.0	97.0
Magnesium^2^, mg	34.7	79.4	229.0	242.0
Vitamin B_3_^2^, mg	2.7	2.3	87.0	94.0
Vitamin B_6_, mg	0.3	0.2	78.0	64.0
Vitamin B_2_^2^, mg	0.4	0.2	53.0	60.0
Vitamin B_1_^2^, mg	0.3	0.2	69.0	72.0
Vitamin A, μg	80.0	128.0	161.0	150.0
Vitamin B_12_, μg	0.7	0.5	77.0	89.0
Vitamin C^2^, mg	9.0	11.0	122.0	130.0
Zinc (mg)	1.5	2.1	140.0	125.0

Abbreviation: HCM, hot cooked meal.

1 Nutrient content of the HCM as a percentage of the guidelines after replacing egg with milk/curd.

2 Represents one-third of the estimated average requirement.

**FIGURE 1:**
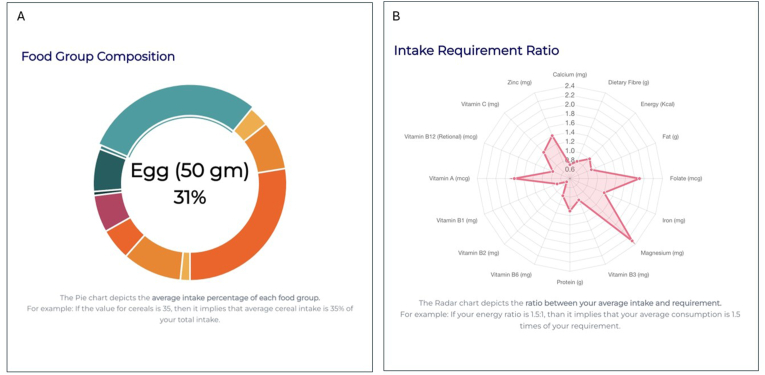
(A) Each segment in the doughnut diagram represents a particular food group and the proportionate allocation of that food group in the optimized HCM expressed as percentage of total weight of optimized foods. Details are displayed when the cursor hovers on a section, when using the online ICDS-SNP tool (https://www.datatools.sjri.res.in/SNP/). (B) Radar chart representing the ratio of quantity of different nutrients in the optimized HCM to their recommended values, such that the line corresponding to 1 represents the optimized nutrient value meeting the recommended value of nutrient intake in HCM giving a ratio of 1. HCM, hot cooked meal; ICDS, Integrated Child Development Services; SNP, Supplementary Nutrition Program.

The proportionate allocation of optimized HCM to different foods groups is shown in Figure 1A, and a radar chart was used to represent the ratio of nutrient in the optimized solution to their recommendations for HCM (Figure 1B). The addition of animal foods like milk and milk products, in a snack or in the main meal, would help meet these gaps but at an additional cost of ₹1.5 per child per day. Subsidized milk and curd could help meet all the nutrient recommendations (Table 2) through the HCM.

**FIGURE 2:**
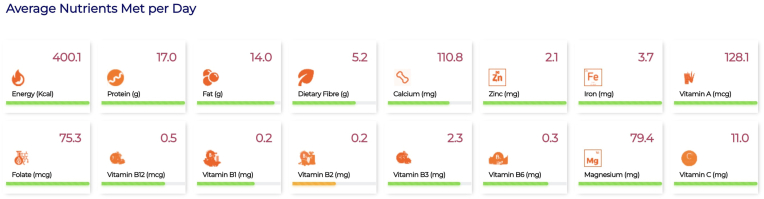
The adequacy of the daily average nutrient intake supplied in the optimized hot cooked meal against its recommended content is depicted through a color-coded system. The color green represents the condition when the nutrient content is >60% of its recommended value; the color orange indicates a content of 30% to 60% of the recommended value, and the color red indicates a content <30% of the recommended value.

The weekly nutrient provision analysis is shown as a line chart (Supplemental Figure 2), showcasing variations in macronutrient intake throughout the week, along with their fat:energy and protein:energy ratios. The advanced analytics from the combination of foods, such as food group contributions, toward quantity of nutrients in the optimized solution and the proportionate cost of each food group are given in Supplemental Figure 3. Food group-wise ingredient contributions to the optimized solution are displayed using stacked bar diagrams (Supplemental Figure 4). These visualizations assist users in understanding which food groups contribute most to each nutrient in the optimized solution and which ingredients contribute most to the cost of the optimized menu. In this instance, the adequacy of the average nutrient intake per day for the HCM is represented using 3 colors, where green indicates meeting >60% of the recommended value, orange signifies meeting 30% to 60% of the recommended value, and red indicates meeting <30% of the recommended value (Figure 2). In the suggested menu for Karnataka, >60% of the recommended value was met by the optimized solution for all nutrients except vitamin B_2_.

**FIGURE 3:**
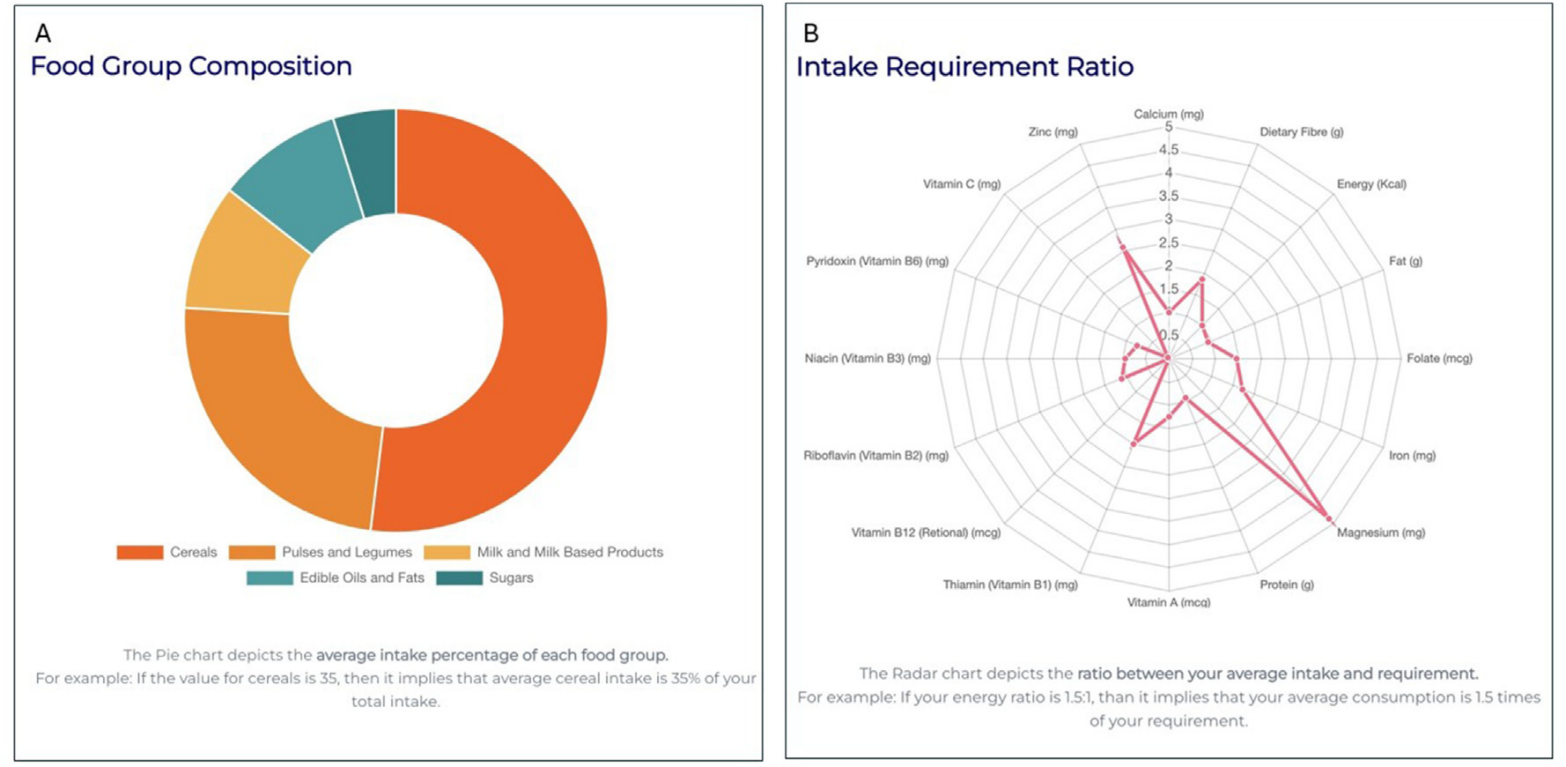
(A) Each segment in the doughnut diagram represents a particular food group and the proportionate allocation of that food group in the optimized THR expressed as percentage of total weight of optimized foods. Details are displayed when the cursor hovers on a section, when using the online ICDS-SNP tool (https://www.datatools.sjri.res.in/SNP/). (B) Radar chart representing the ratio of quantity of different nutrients in the optimized THR to their recommended values, such that the line corresponding to 1 represents the optimized nutrient value meeting the recommended value of nutrient intake in THR giving a ratio of 1. ICDS, Integrated Child Development Services; SNP, Supplementary Nutrition Program; THR, take home ration.

**FIGURE 4:**
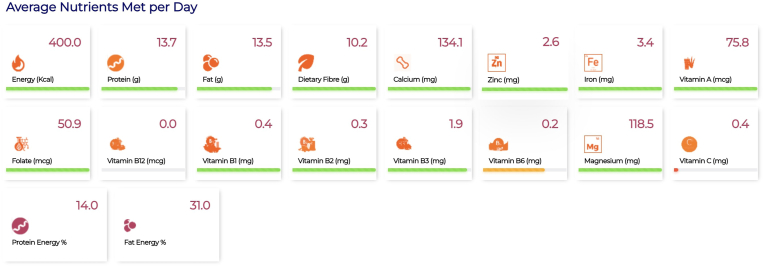
The adequacy of the daily average nutrient intake supplied in the optimized take home ration against the recommendation is depicted through a color-coded system. The color green represents the condition when the nutrient content is >80% of its recommended value; the color orange indicates a content of 50% to 80% of the recommended value, and the color red indicates a content <50% of the recommended value.

Similarly, the tool was used to optimize a THR product for 1- to 3-y-old children in Karnataka. The tool was first used to optimize a THR product using retail market prices collected from Karnataka and using subsidized prices (for example, milk powder with a subsidized price of ₹259/kg was considered). Cost calculations are provided for an individual’s daily, weekly, monthly, and yearly requirements in Supplemental Table 3 for the optimized THR solution, and Supplemental Table 4 shows their ingredient composition. Table 3 provides the nutrient composition of the optimized THR solution. The allocation of optimized THR to different food groups is shown in Figure 3A, and a radar chart was used to represent the ratio of nutrients in the optimized solution to their recommendations in Figure 3B. For THR optimization, analytics similar to that of HCM is given in Figure 4.

**TABLE 3:** Nutrient composition in an example of an optimized THR for children aged 1 to 3 y in the Indian state of Karnataka

Nutrient	Gazette of India guidelines	Nutrient composition	Percentage of guidelines met
Energy, kcal	400.0	400.0	100.0
Protein, g	15.0	13.7	91.3
Fat, g	15.0	13.5	90.0
Calcium, mg	135.0	134.1	99.3
Dietary fiber^1^, g	5.6	10.2	183.8
Folate, μg	35.0	50.9	145.4
Iron, mg	2.0	3.4	170.0
Magnesium^1^, mg	24.3	118.5	487.1
Vitamin A, μg	60.0	75.8	126.3
Vitamin B_1_^1^, mg	0.2	0.4	200.0
Vitamin B_12_, μg	0.3	0.0	0.0
Vitamin B_2_^1^, mg	0.3	0.3	111.1
Vitamin B_3_^1^, mg	2.0	1.9	95.0
Vitamin B_6_, mg	0.3	0.2	74.1
Vitamin C^1^, mg	8.0	0.4	5.0
Zinc, mg	1.0	2.6	260.0

Abbreviation: THR, take home ration.

1 Represents one-third of the estimated average requirement.

The tool is meant to empower users to create tailored menus specific to their region, aligning with the preferences of beneficiaries at the selected Anganwadi. For instance, when a user selects the state Karnataka, the foods that are available in Karnataka state alone are listed and their local names in regional languages are also provided. Subsequently, the tool provides recipes based on user-selected ingredients, allowing users to opt for familiar culinary options. Users have the option of performing multiple optimizations in separate windows and compare and analyze different weekly menus and THR products to make informed decisions on the final combination. To facilitate easy access, a comprehensive user manual with video tutorials has also been provided for the publicly available web tool (https://www.datatools.sjri.res.in/SNP/).

## Discussion

This article describes a general diet optimization model using LP that can fulfill the contextual needs for any supplementary feeding program, provided that a database of locally available foods, their nutritive values, and their costs is available. In this report, the tool that was created for the ICDS-SNP program of India and specifically applied to children aged 1 to 6 y is described.

The optimization algorithm by itself is not trivial; it is intensive and requires expert knowledge in nutrition as well as advanced programming capability to create. Rules for the provision of food supplements can be difficult to fulfill, as these are created to fill gaps, sometimes with a single meal, in an otherwise deficient diet. An example is the rules set by the ICDS-SNP [3], which are difficult to execute because the SNP protein and fat content have been set at 15 to 20 g each, while the energy is maintained at 400 kcal. It is not easy to arrive at food combinations that align with these recommendations at minimal cost. Hence, the optimization model has been developed into a tool that can be easily used by implementing officials responsible for making decisions on the procurement of SNP provisions, at the allowable budget, in this case, ₹8 per child per day. There is an effort to optimize a diverse diet, but sometimes this may not be possible at the available budget and alternate strategies to meet the macro- and micronutrient recommendations must be explored. This could include additional subsidies on some of the quality food ingredients such as egg and milk, or even additional budgetary allocations such that nutrient recommendation can be met through diverse diet, which is represented as a doughnut diagram in the tool. Alternative, state-specific nutrient supplements can be added to the ICDS-SNP based on the quantity of micronutrients that are unmet by the optimized meal and this would be minimal inclusion. This information is vital to policy makers. In general, while it is easy to meet iron, zinc, folate, magnesium, and vitamin A recommendations, meeting calcium and B vitamins recommendations requires the addition of green leafy vegetables, milk, and egg in adequate quantities.

The application of LP in nutrition is pivotal in optimizing diets and food formulations to meet nutritional needs efficiently and cost-effectively. A study on Japanese adults used LP to create nutritionally optimal food patterns that adhere to dietary reference intakes while incorporating typical Japanese foods, demonstrating the significant dietary adjustments needed, especially among younger individuals to increase fruit, vegetable, and whole grain consumption while reducing salt intake [9]. Extending LP from diet optimization to food formulation, another study addressed the challenges of creating an affordable, nutritious porridge mix for emergency situations in rural Mozambique, illustrating the difficulty of using only local ingredients due to the high costs of mineral-rich foods, but achieving success with mineral supplements [10]. Similarly, LP was used to design new formulations of ready-to-use therapeutic food for Ethiopian children with SAM, leveraging local ingredients to create low-cost, nutrient-compliant formulations that were validated in laboratory tests [11]. Additionally, the capability of LP was highlighted in formulating complementary feeding diets using local foods, providing a more rigorous and efficient alternative to the traditional trial-and-error method and facilitating the development of affordable, nutritionally adequate diets [12]. Finally, a study aimed at developing complementary feeding recommendations for Indonesian infants used a 4-phase LP and goal programming approach, identifying critical nutrient gaps and recommending specific local foods and fortified products to enhance dietary quality [13]. The diet optimization tools that are currently available globally need ways to add new databases or allow the inclusion of regional programmatic rules. They certainly do not consider the recommendations of the ICDS-SNP. These studies collectively underscore the effectiveness of LP in addressing nutritional challenges by tailoring solutions to specific populations and regional food availabilities. For example, the Cost of the Diet method and software [14] were created to employ LP for a more profound comprehension of how poverty might influence an individual’s ability to meet their dietary requirements. Optifood [15], on the other hand, offers insights into the optimal combinations of local foods to maximize nutrient intake. It can objectively assess whether these combinations can provide nutritionally sufficient diets for an entire population. However, Optifood is a commercial software and is not built to the specifications of the ICDS-SNP program or any regional requirement. Hence only a trained nutritionist who has insights of local diet patterns and nutrient gaps can use the software to get relevant outputs, but not the implementing officials who are usually bureaucrats entrusted with the responsibility of procuring raw food provisions.

The ICDS-SNP optimization tool that is described here, was developed with support from the WHO India Office (https://www.datatools.sjri.res.in/SNP/). It has compiled and standardized a comprehensive collection of recipes from each State in India, which were primarily collected for the purpose of the optimization model and the tool from *Anganwadis* and beneficiaries. This framework helps the user to have customized optimizations to suggest SNP provisions that align with the dietary preferences of the population that they cater to. What sets this tool apart from existing dietary software is its flexibility and user-friendly features that permits users to customize the optimization at an *Anganwadi* (or any such center) or at population levels such as district or state level, using very simple navigable options. The tool has been designed with user interfaces that are readily comprehensible and multiple food combinations and optimizations can be explored within seconds (maximum of 1 minute) as all the complex calculations of extracting the nutritive values of different foods for multiple macro and micronutrients, and performing the complex optimization algorithm, is performed efficiently at the backend. Moreover, the tool demonstrates that by introducing subsidies for certain ingredients, it becomes possible to achieve a nutrient-rich and diverse diet without resorting to unnecessary fortification.

There are some limitations to this tool. The data collected for the development of tool was not random and hence an exhaustive list of diets of children in a state is not available. However, there is provision to add foods and recipes to the database used in the tool for a logged in user and the tool is not limited by the data collected. It does not factor in nutrient losses that can occur during the cooking process, which would range from 10% to 60% for vitamins, depending on the cooking process [16]. There may also be instances where the time required to arrive at optimized solutions exceeds the one-minute timeline. For some states, the ICDS-SNP program run by the government may be limited in its ability to include quality foods such as vegetables, fruits, and animal source food due to problems of affordability, availability, and accessibility. This would reflect in the optimized solutions generated as not all micronutrient recommendations may be met by the optimized meal plan. This can prompt the user, who would be a government official responsible for the program, to identify strategies to improve the nutritional quality of HCMs and THRs. On the other hand, there is potential for such optimization tools to be applied to other programs, like mid-day meal programs and other feeding initiatives designed to benefit different kinds of beneficiaries in the future. The suggested ICDS-SNP optimization tool is a valuable resource for policy makers, implementers, nutritionists, and researchers, enabling them to inform decisions on provisions for specific populations based on a database of local dietary preferences. The tool can also help compare various options for SNPs and identify foods that need to be subsidized, and by what amount, to meet the nutrient recommendations using diverse diets.

## Author contributions

The authors’ responsibilities were as follows – TT, RG, HSS, AVK: conceptualization; FA, JRM, AM, GB: methodology; AA, AKM: software; MSN, SA, PM, LV, RM, SAB, SG-J, VN, HG, SZQ: data collection, validation, and manuscript review; FA, JRM, AM, GB, JRM, TT: formal analysis; FA, JRM: data curation; FA, JRM, TT: original draft preparation,; FA, JRM, RG, HSS, AVK, TT: draft review and editing; JRM, AA, AKM, FA: visualization development; TT: supervision; and all authors: read and approved the final manuscript.

## Conflict of interest

RG is an employee of WHO. All other authors report no conflicts of interest.

## Funding

The study was funded by WHO (India Office), Grant Number 202868487-2.

## Data availability

Data described in the manuscript, code book, and analytic code will be made available upon reasonable request.
