# Supplementary material for: An Optimization Tool to Formulate Diets within a Supplementary Nutrition Program for Children
Source: Curr Dev Nutr. 2024 Jun 29;8(7):104409. doi: 10.1016/j.cdnut.2024.104409 (PMC11305195; doi:10.1016/j.cdnut.2024.104409)
Supplement: Multimedia component 1 [file mmc1.pdf]

An Optimisation Tool to Formulate Diets within a Supplementary Nutrition Program for Children:
Fathima Ayoob, Jawahar R Manivannan

Supplementary Tables and Figures

Supplementary Table 1. Fortified Foods per 100 grams in ICDS and their fortification level.

**Nutrients**	Rice	Whole Wheat Flour	Milk	Salt
Iron (mg)	4.25	4.25		85.0
Folic Acid (mcg)	12.5	12.5		
Vitamin B_{12} (mcg)	0.125	0.125		
Vitamin A (mcg)			75.0	
Iodine (ppm)				15.0

Supplementary Table 2. Optimised six-day menu for Hot Cooked Meal with market survey prices for a child 3-6y in Karnataka.

Day	Type of Meal	Recipe Name	Minimum Serving Size	Unit	Cost per Beneficiary (Rs.)	Subsidised Cost per Beneficiary (Rs.)
1	Main meal	Steamed rice	1.28	Bowl	0.17	0.17
		Egg	1.0	No.	5	2.5
		Amaranthus horse-gram dal	0.64	Bowl	3.13	3.13
	Snack	Groundnut bar	10.0	gm	1.2	1.2
2	Main meal	Steamed rice	1.32	Bowl	0.17	0.17
		Egg	1.0	No.	5	2.5
		Green gram dal curry	0.66	Bowl	3.13	3.13
	Snack	Roasted groundnut	10.0	gm	1.2	1.2
3	Main meal	Steamed rice	1.31	Bowl	0.17	0.17
		Egg	1.0	No.	5	2.5
		Horse-gram dal sambar	0.65	Bowl	3.14	3.14
	Snack	Roasted groundnut	10.0	gm	1.2	1.2
4	Main meal	Steamed rice	1.34	Bowl	0.17	0.17
		Egg	1.0	No.	5	2.5
		Sprouted horse-gram dal sambar	0.67	Bowl	3.18	3.3
	Snack	Roasted groundnut	10.0	gm	1.2	1.2

5	Main meal	Egg	1.0	No.	5	2.5
		Vegetable pulao	0.8	Bowl	2.95	2.95
	Snacks	Wheat rava payasam	0.5	Bowl	1.85	1.85
6	Main meal	Steamed rice	1.3	Bowl	0.17	0.17
		Egg	1.0	No.	5	2.5
		Horse gram dal fenugreek leaves sambar	0.65	Bowl	3.47	3.63
	Snacks	Roasted groundnut	10.0	gm	1.2	1.2
Total cost per week (6 days)					57.7	43.0
Total cost per day					9.6	7.16

Supplementary Table 3. Cost of an example optimized Take Home Ration solution for children 1-3y from Karnataka state of India.

	**Cost**	**Subsidised Cost**
Per day (₹, average)	7.8	6.7
Per week (₹, 6 days)	46.8	40.1
Per month (₹, 25 days)	194.9	167.2
Per year (₹, 300 days)	2339.3	2006.3

Supplementary Table 4. Ingredient composition of the optimized THR solution with market and subsidised prices**.**

	**Wheat Flour**	**Whole Green Gram**	**Whole Milk Powder**	**Fortified Oil**	**Sugar**	**Total**
Quantity/beneficiary/day (g)	54	25	10	10	5	104
Cost/beneficiary/day (₹)*	0.11	2.04	3.7	1.75	0.2	7.8
Subsidised cost/beneficiary/day (₹)*	0.11	2.04	2.59	1.75	0.2	6.69
Cost/kg (₹)*	2	84	370	180	40	
Subsidised cost/kg (₹)*	2	84	259	180	40	

* 82 Indian rupee (₹) equivalent to 1 USD

Supplementary Figure 1. Sampling strategy for data collection

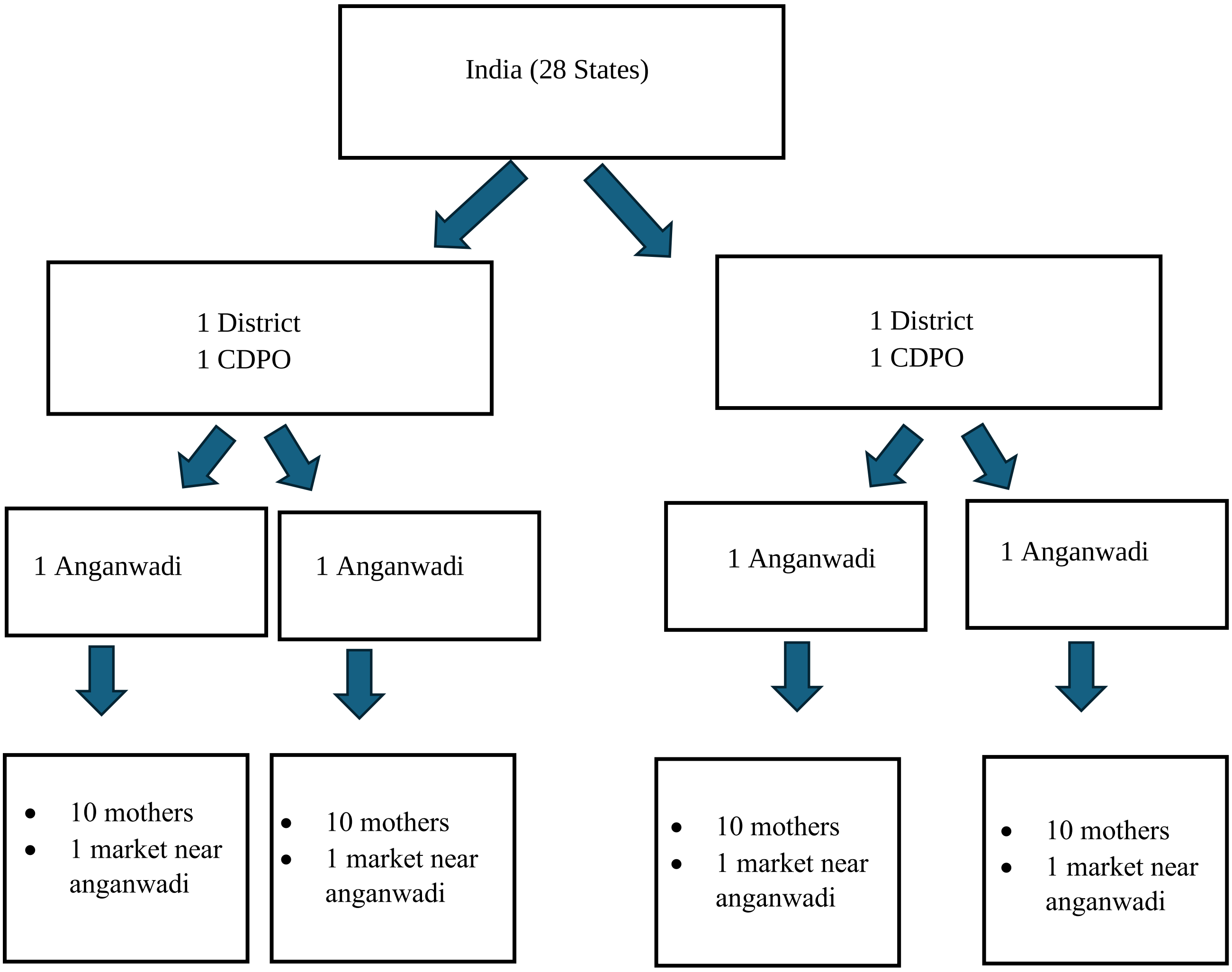

Supplementary Figure 2: Line chart illustrating the weekly analysis of nutrient provision, highlighting fluctuations in macronutrient composition in each day, along with the corresponding fat:energy and protein:energy ratios for an optimized solution for Hot Cooked meal in Karnataka state.

Weekly Nutrient Analysis

DAY	ENERGY	PROTEIN	FAT	PE RATIO	FE RATIO
1	400 Kcal	18.0 g	13.6 g	18.0	31.0
2	400 Kcal	17.7 g	14.0 g	18.0	31.0
3	400 Kcal	16.8 g	14.2 g	17.0	32.0
4	400 Kcal	17.3 g	13.8 g	17.0	31.0
5	400 Kcal	17.8 g	14.0 g	18.0	31.0
6	401 Kcal	14.2 g	14.4 g	14.0	32.0

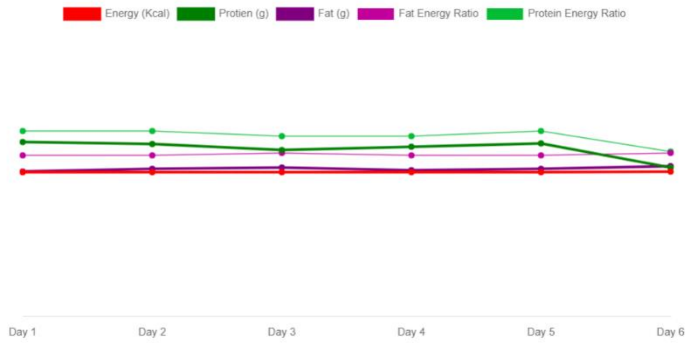

Supplementary Figure 3: Stacked bar diagrams of the percent contribution of each food group to macro and micro-nutrient content in the example of optimized Hot Cooked Meal for 3-6y children in Karnataka. Each bar represents the 100% of the nutrient in the optimized HCM and each colour within that bar is a % contribution of a food group to that nutrient.

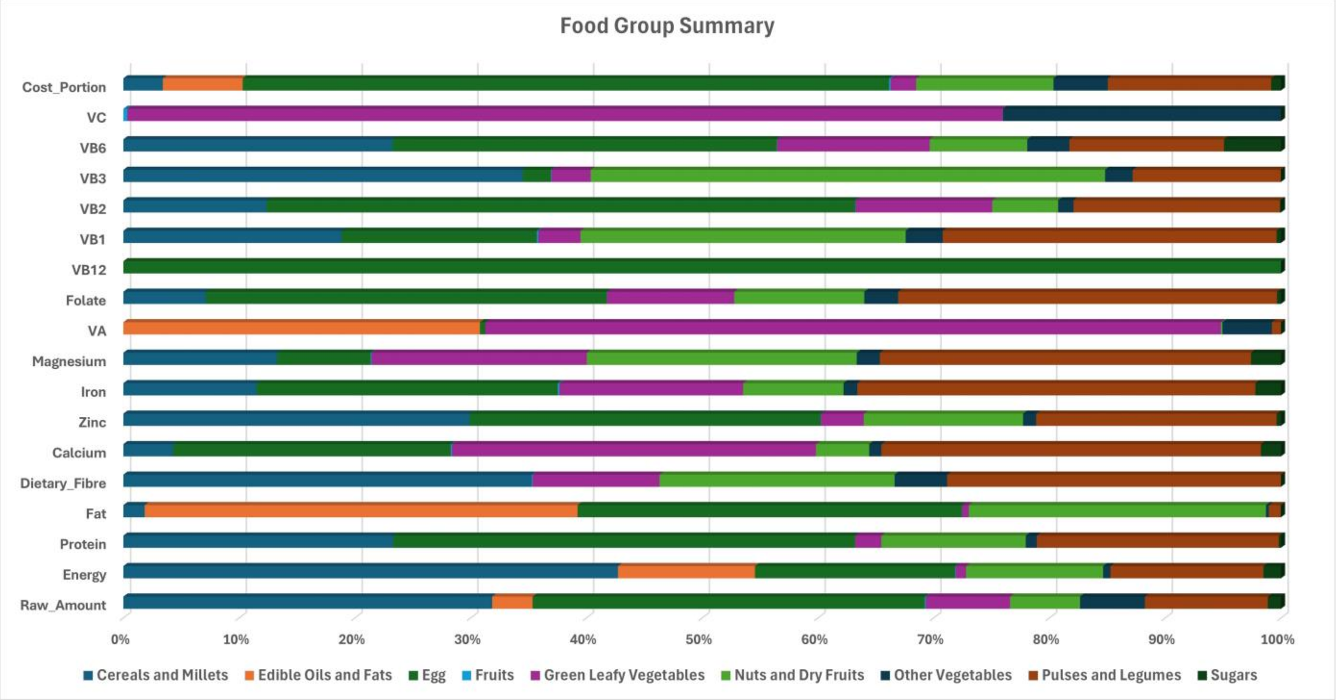

Supplementary Figure 4: Stacked bar diagram of the percent contribution of each food to macro and micro-nutrient content in the example of optimized Hot Cooked Meal for 3-6y children.

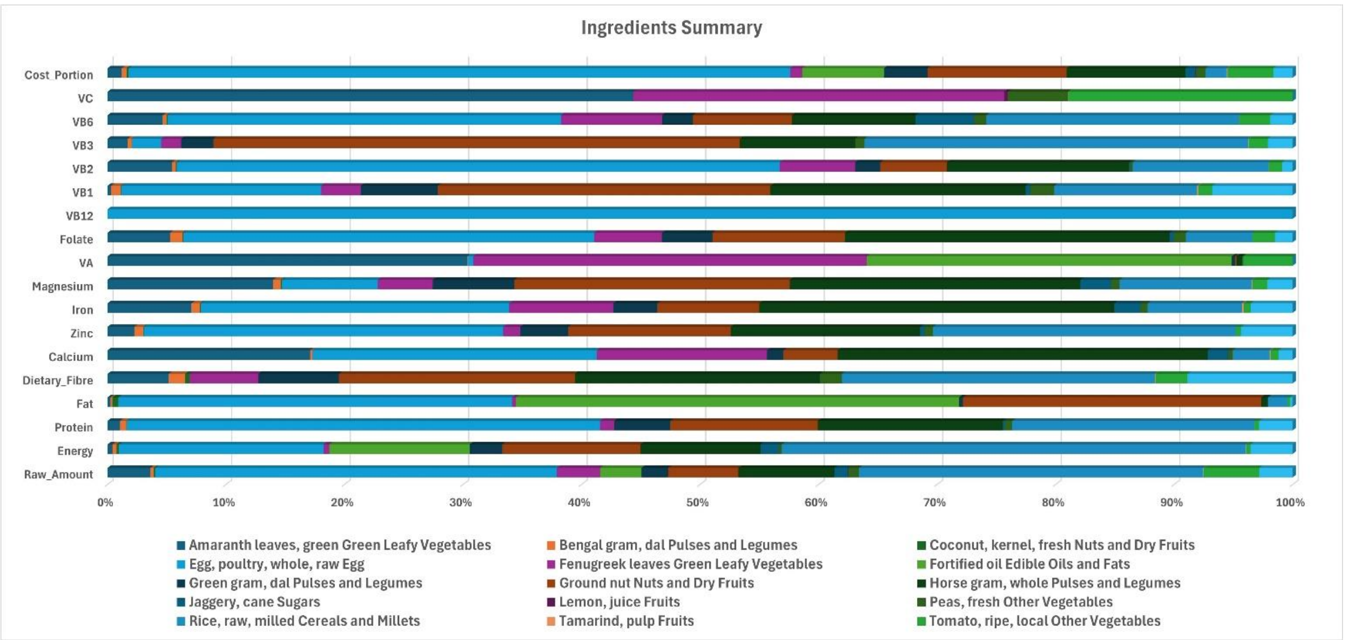

LPP of HCM

Objective function

$$\min \sum_{i=1}^{I} S_i C_i \qquad (1)$$

Subject to

Constraint for EAR and TUL of nutrients

$$EAR\ (N_j) \leq \sum_{i=1}^{I} S_i N_{ij} \ \leq \ TUL\ (N_j) \qquad , \forall i \in Recipe, \forall\ j \in Nutrients$$

Constraints for Normal children

$$\sum_{j=1}^{J} S_j D_j \ \leq \ 1.5\ , \forall j \in Main\ dish\ recipes$$

$$\sum_{j=1}^{J} D_j \ = \ 1\ , \forall j \in Main\ dish\ recipes$$

$$\sum_{i=1}^{I} S_i D_i \ \leq \ 1\ , \forall i \in Snack\ recipes$$

$$\sum_{i=1}^{I} D_i \ = \ 1\ , \forall i \in Snack\ recipes$$

Constraints for SAM children

$$\sum_{j=1}^{J} S_j D_j \ \leq \ 1.5\ , \forall j \in Main\ dish\ recipes$$

$$\sum_{j=1}^{J} D_j \ = \ 1\ , \forall j \in Main\ dish\ recipes$$

$$\sum_{i=1}^{I} S_i D_i \ \leq \ 2\ , \forall i \in Snack\ recipes$$

$$\sum_{i=1}^{I} D_i \ = \ 1\ , \forall i \in Snack\ recipes$$

Constraints for proportions in combinations, where R_1 (main dish), R_2 (curry) and R_3 (side dish)

$0.5\ S_i D_i \ \leq \ S_k D_k \ \leq \ 0.8\ S_i D_i \ , \forall i \in R_1, \forall\ k \in R_2$

$0.25\ S_i D_i \ \leq \ S_m D_m \ \leq \ 0.75\ S_i D_i \ , \forall i \in R_1, \forall m \in R_3$

Constraint for compulsory foods

$0.5 \leq S_i D_i \ \leq \ 1.5\ , \forall\ i\ in\ Compulsory\ foods$

$where\ D_i = \ 1\ , \forall\ i\ in\ Compulsory\ foods$

Constraint for additional foods

$S_i D_i \ \leq \ 1.5\ , \forall\ i\ in\ Additional\ foods$

where $D_i \in \{0,1\}, \forall\ i\ in\ Additional\ foods$

$\boldsymbol{S_i, C_i, N_{ij}, N_j, S_k, S_m} \in \mathbb{R}^+$

where S is the serving size of the corresponding recipe ~~type~~, , C_i is the cost of the ith recipe and N_{ij} is the jth nutrient value of the ith recipe and N_j is the jth nutrient value.

$\boldsymbol{D_i, D_j, D_k, D_m} \in \{\boldsymbol{0, 1}\}$

where D are the decision variables for the corresponding recipe ~~type~~, i.e whether the recipe shall be included in the model or not.

LPP of THR

Objective function

$$min \sum_{i=1}^{I} Q_i C_i \quad (2)$$

subject to

$$EAR\ (N_j) \le \sum_{i=1}^{I} Q_i N_{ij} \ \le \ TUL\ (N_j) \qquad , \forall i \in Food\ Items, \forall j \in (Energy, Protein\ and\ Fat)$$

Constraint on the inclusion of at least one food item from each food group for whole foods and blended premix

$$N_{k\_min} \ \le \sum_{i=1}^{I} D_{ik} \ \le \ N_{k\_max} \quad , \forall i \in Items\ in\ k^{th}\ food\ group$$

Constraints on the minimum and maximum quantities within each food group for whole foods and blended premix

$$Q_{k\_min} \ \le \sum_{i=1}^{I} Q_{ik} \ \le \ Q_{k\_max} \quad , \forall i \in Items\ in\ k^{th}\ food\ group$$

Constraints subjected to quantity restrictions at the food item level for whole foods and blended premix

$$D_i Q_{i\_min} \ \le Q_i \ \le D_i \ Q_{i\_max} \quad , \forall\ i \in \{oils, eggs, ground\ nut, sugar, jaggery, whole\ milk\ powder, black\ \&\ white\ sesame\ seeds\}$$

Constraint for maintaining a cereal:pulse ratio of 2:1

$$1.8 \sum_{m=1}^{M} Q_m \ \le \sum_{n=1}^{N} Q_n \ \le \ 2.2 \sum_{m=1}^{M} Q_m \ , \forall m \in Pulses\ and\ \forall\ n \in Cereals$$

Constraint for compulsory inclusion of cereal or millet

$$Q_{k\_min} \ \le \sum_{i=1}^{I} Q_{ik} \ \le \ Q_{k\_max} \quad , \forall i \in Items\ in\ cereals\ or\ millets$$

$\boldsymbol{Q_i, C_i, N_j, N_{ij}, Q_{k\_min}, Q_{k\_max}, Q_{ik}, Q_{i\_min}, Q_{i\_max}, Q_m, Q_n} \in \mathbb{R}^+$

where Q_i is the quantity of the ith food item, C_i is the cost of the ith food item, N_j is the jth nutrient value, N_{ij} is the jth nutrient value of the ith food item, Q_{k_min} is the minimum quantity of kth food group, Q_{k_max} is the maximum quantity of the kth food group, Q_{ik} is the quantity of ith food item belonging to k[th] food group, Q_{i_min} is the minimum quantity of ith food item, Q_{i_max} is the maximum quantity of the ith food item, Q_m is the quantity of pulses, , Q_n is the quantity of cereals

$\boldsymbol{D_i} \in \{\mathbf{0}, \mathbf{1}\}$

where D_i is the decision variable for the ith food item, i.e whether the food item shall be included in the model or not.

$\boldsymbol{N_{k\_min}, N_{k\_max}} \in \boldsymbol{Q^+}$

N_{k_min} is the minimum number of food items to be chosen from k[th] food group, N_{k_max} is the maximum number of food items to be chosen from k[th] food group.
